# Supplementary material for: Identification of Freezing-Responsive microRNAs and Their Targets in Chinese Jujube by Small RNA and Degradome Sequencing
Source: Int J Mol Sci. 2026 Sep 4;27(17):7912. doi: 10.3390/ijms27177912 (PMC13566890; doi:10.3390/ijms27177912)
Supplement: Supplementary file 1 [file ijms-27-07912-s001.zip › Supplementary Material S2/novel_miR_171.pdf]

| | | |
|---|---|---|
| Provisional ID | : | novel_miR_171 |
| Score total | : | 0.5 |
| Score for star read(s) | : | -1.3 |
| Score for read counts | : | 0 |
| Score for mfe | : | 0.2 |
| Score for randfold | : | 1.6 |
| Score for cons. seed | : | |
| Total read count | : | 360 |
| Mature read count | : | 360 |
| Loop read count | : | 0 |
| Star read count | : | 0 |

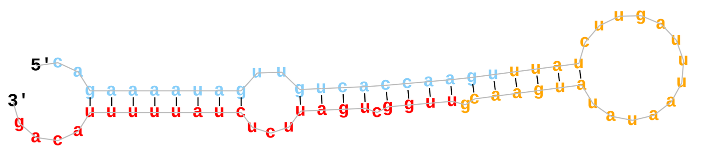

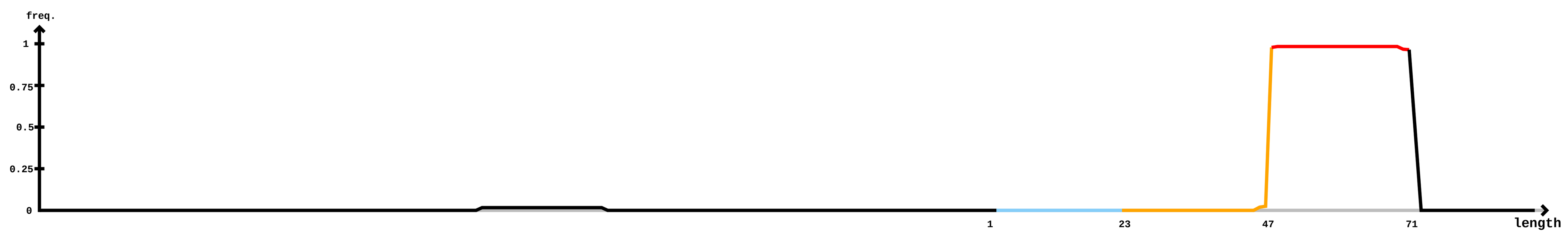


Star Mature

5'- cuugacaaguuacauuacgugaaugaauuauuuguuuaugguaaugaguuuucagauuaguuagucaucuuacaacucuuuaaaauucguuaugauugcuaaaaaugucaaugccaugucuacuuugauuuguuaaaaguuccauucaguaaggccuuucagaaaauaguugucaccaaguuuaucuugauuuaauauaugaacguuggcugauucucuauuuuuacaggcaaugaauggucaauaucuuu -3' exp

.((((((((((....(((((..(((....((((...((.((((((((((..(((...((.((....)).))...)))..))))))))))).))...))))....)))....)))))......)))))))))).....(((((((.....((((....((((((((..(((((((((((((............)))))).)))).))))...))))))))..)))).)))))))..........

| | reads | mm | sample |
|---|---|---|---|
| caacucuuuaaaauucguuaug | 6 | 0 | LCK-3 |
| cguuggcugauucucuauuuuuacag | 1 | 0 | JCK-2 |
| cguuggcugauucucuauuuuuacag | 3 | 0 | LT-1 |
| cguuggcugauucucuauuuuuacag | 1 | 0 | JCK-3 |
| cguuggcugauucucuCuuuuuacag | 2 | 1 | JCK-3 |
| guuggcugauucucuauuuuuacag | 2 | 0 | LT-3 |
| uuggcugauucucuauuuuuac | 3 | 0 | JCK-3 |
| uuggcugauucucuauuuuuac | 1 | 0 | LT-3 |
| uuggcugauucucuauuuuuac | 2 | 0 | JCK-2 |
| uuggcugauucucuauuuuuaca | 1 | 0 | LT-1 |
| uuggcugauucucuauuuuuacag | 4 | 0 | JT-2 |
| uuggcugauucucuauuuuuacag | 3 | 0 | JT-1 |
| uuggcugauucucuauuuuuacag | 40 | 0 | JCK-1 |
| uuggcugauucucuCuuuuuacag | 1 | 1 | LCK-3 |
| uuggcugauucucuauuuuuacag | 19 | 0 | LT-3 |
| uuggcugauucucuauuuuuacag | 110 | 0 | LCK-3 |
| uuggcugCuucucuauuuuuacag | 1 | 1 | LCK-3 |
| uuggcugauucucuauuuuuacag | 34 | 0 | LT-1 |
| uuggcugauucucuauuuuuacag | 8 | 0 | JT-3 |
| uuggcugauucucuauuuuuacag | 43 | 0 | LT-2 |
| uuggcugauucucuauuuuuacag | 40 | 0 | JCK-2 |
| uuggcugauucucuauuuuuacag | 39 | 0 | JCK-3 |
| uggcugauucucuauuuuuacag | 1 | 0 | JCK-1 |
| uggcugauucucuauuuuuacag | 1 | 0 | JCK-3 |
